# Acceptable range of timing error at bat-ball impact in baseball depends on the bat swing path

**DOI:** 10.3389/fspor.2025.1557145

**Published:** 2025-03-28

**Authors:** Hirotaka Nakashima, Gen Horiuchi, Arata Kimura, Shinji Sakurai

**Affiliations:** ^1^ Department of Sport Science and Research, Japan Institute of Sports Sciences, Tokyo, Japan; ^2^ Graduate School of Health and Sport Sciences, Chukyo University, Aichi, Japan; ^3^ College of Sport and Health Science, Ritsumeikan University, Shiga, Japan; ^4^ Faculty of Sports and Health Studies, Hosei University, Tokyo, Japan; ^5^ School of Health and Sport Sciences, Chukyo University, Aichi, Japan

**Keywords:** batting, hitting, swing plane, impact zone, ball trajectory

## Abstract

**Introduction:**

In baseball, a common instruction emphasizes aligning the bat swing trajectory with the pitched ball trajectory near impact when viewed from the side. This alignment is believed to widen the acceptable range of timing error, thereby enhancing batting average. While prior studies have explored the effects of swing speed and sweet spot contact on batted ball velocity, the specific influence of bat swing path on the acceptable range of timing error during bat-ball impact has not been adequately investigated. We aimed to quantify the acceptable range of timing error and to investigate the swing characteristics that influence this range.

**Methods:**

Eighteen pitched ball trajectories thrown by 10 collegiate pitchers and 145 bat swing trajectories performed by 29 collegiate batters were acquired in independent experimental settings. From these trajectories, the acceptable time and distance ranges of timing error, in which the ball could be impacted by the bat's sweet spot, were calculated.

**Result and discussion:**

The average acceptable range of timing error was 9.36 ± 6.25 ms in time and 0.227 ± 0.163 m in distance. However, these ranges varied significantly (time: 2.48–30.40 ms; distance: 0.056–0.614 m) depending on the specific swing trajectory. Furthermore, our findings revealed that the acceptable range of timing error is not solely determined by a single swing characteristic but rather by the interplay of multiple factors, including the bat swing trajectory as viewed from the side and above and the bat angle at impact. These results suggest a need for a multifaceted approach to swing instruction, considering these inter-related factors to optimize a batter's ability consistently to make solid contact.

## 1 Introduction

The official baseball rule clearly states, “The objective of each team is to win by scoring more runs than the opponent” (1). In other words, batters are required to make hits and score runs. Statistical findings from Major League Baseball games show that batted ball velocity strongly correlates with the batting average, which in turn influences the average runs scored per game (2). Thus, batted ball velocity is a critical factor in determining a baseball batter's performance. Previous studies have demonstrated that increasing bat swing speed and impacting the ball at the bat's sweet spot significantly enhance batted ball velocity (3–5), and methods for achieving higher batted ball velocity are well established (6–8).

**Figure 1:**
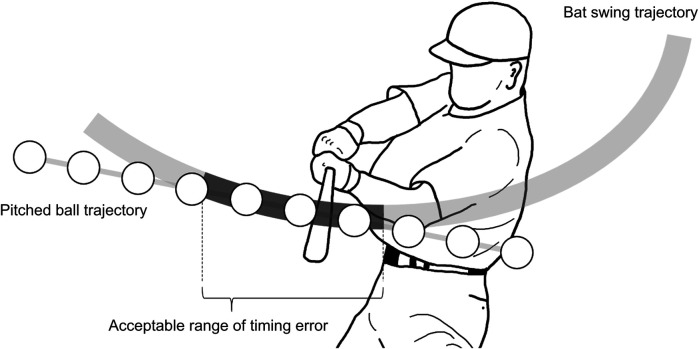
Acceptable range of timing error at bat-ball impact.

However, baseball batting is widely regarded as one of the most challenging tasks in sports (9) due to severe spatiotemporal restrictions. Batters must consistently hit the ball at a narrow range of sweet spots, approximately 10 cm along the long axis and 2 cm along the short axis of the bat (10), while reacting to pitched balls of varying speeds and trajectories. In addition, pitched balls typically reach home plate in approximately 400–600 ms, yet the time from the initiation of the forward swing to the bat-ball impact is approximately 130–280 ms (11–13). Moreover, batters experience a delay of approximately 150–250 ms between decision-making and initiating the forward swing (14–16). Given these constraints, batters must decide whether to swing within an exceptionally short time. To make matters more challenging, the acceptable range of timing error at bat-ball impact (Figure 1) is about 10 ms (17). This implies that an accurate bat-ball impact cannot be achieved if the batter starts to swing 5 ms early or late.

To address these challenges in baseball practice, the bat swing trajectory must align with the pitched ball trajectory to increase the acceptable range of timing error (Figure 1). Williams and Underwood (18) suggested that batters should swing slightly upward to align with the downward trajectory of the pitched ball near the home plate, which is believed to widen the acceptable time and distance ranges of timing error. This instruction is generally carried out (19). Although previous study (17) quantified the acceptable range of timing error through experiment using a pitching machine, no study has quantified the range by considering the fact that pitched ball and bat swing trajectories differ for each pitch and swing. Thus, it has not been elucidated how long or how far the acceptable range of timing error can be widened by modifying bat swing trajectories. A good starting point is to quantify the acceptable range of timing error and describe the extent to which modifying the bat swing trajectory can widen the acceptable range of timing error.

Coaches and analysts typically observe the bat swing trajectories from a side view (20) (Figure 1). The acceptable range of timing error would be maximized when the pitched ball trajectory aligns with the bat sweet spot trajectory in a side view. However, this assumption may not always be true because the bat swing involves rotational movement around the grip, and the vertical bat angle varies based on the height and course of the pitched ball. Therefore, relying solely on side-view observations may not enhance performance. A three-dimensional interpretation of the acceptable range of timing errors is essential for improving batting performance.

We aimed to address these gaps. Specifically, its objectives were twofold: first, to quantify the acceptable time and distance ranges of timing error at bat-ball impact in baseball; second, to explain the swing characteristics that influence the acceptable range of timing error from a three-dimensional perspective.

## 2 Materials and methods

### 2.1 Experimental design and ethical statement

Two separate experiments were conducted to investigate the acceptable range of timing errors during baseball batting. In the first experiment, the pitched ball kinematics of 10 pitchers were recorded during an outdoor pitching session to simulate pitched ball trajectories. In the second experiment, the bat swing trajectories of 29 batters were captured in a laboratory setting. These trajectories were superimposed to calculate the acceptable range of timing errors.

This study adhered to the principles of the Declaration of Helsinki and was approved by the Ethics Committee of Chukyo University (No. 2024-109). Owing to the nature of the study, an opt-out approach was employed. Participants were informed via the Chukyo University website and were given the opportunity to opt out if they did not wish to participate.

### 2.2 Pitching data

#### 2.2.1 Participants

Ten male collegiate baseball pitchers (height: 177.4 ± 4.9 cm, body mass: 77.6 ± 9.6 kg, age: 19.4 ± 0.8 years) participated in this study. All pitchers were right-handed and used either an overhand or three-quarter throwing style, as defined by Miyanishi et al. (21).

#### 2.2.2 Experimental procedure

The pitching experiment was conducted in an outdoor bullpen, a practical area for baseball pitching. After sufficient warmup, participants were instructed to throw fastballs and curveballs if possible. The session continued until a pitch that passed through the strike zone and was satisfactory to the participant was obtained for each pitch type. All participants threw fastballs, while eight threw curveballs, resulting in 18 analyzed pitches. The fastballs exhibited a high degree of hop, while the curveballs had a sharp downward break (22). These pitch types were selected to provide a comprehensive dataset of diverse trajectories.

#### 2.2.3 Data collection

Two high-speed video cameras (Coaching Cam, Sports Sensing Co., Japan) were used to record the linear kinematics of the pitched ball around its release point. One camera (Camera 1) was placed behind the catcher, and the other (Camera 2) in the third base direction. These cameras operated at a frame rate of 240 fps with an exposure time of 1/4,000 s. To capture angular kinematics of the pitched ball immediately after the ball release, a high-speed video camera (Camera 3, Fastec TS3, Asanuma & Co., Japan) was positioned approximately 1 m behind the pitching rubber, aligned with the pitcher's release point, and leveled to ensure accurate measurements. The camera operated at a frame rate of 1,000 fps with an exposure time of 1/5,000 s. The optical axis of the lens was directed toward the home plate.

#### 2.2.4 Data processing

Positional coordinates were extracted from video images using a motion analysis system (Frame DIAS V, Q'sfix Co., Japan).

For linear kinematics, the centers of the balls at and after release in the video images recorded by Camera 1 and Camera 2 were manually digitized, and their three-dimensional coordinates were computed through the direct linear transformation method. The X-, Y-, and Z-axes of the global coordinate system defined the horizontal direction from the third to the first bases, the horizontal direction from the catcher to the pitcher, and the vertical upward direction, respectively. The origin was set at the tip of the home plate. From this data, the release position ($x{\_}_{0}$, $y{\_}_{0}$, $z{\_}_{0}$) and velocity components ($v_{x\_0}$, $v_{y\_0}$, $v_{z\_0}$) of the pitch were calculated.

For angular kinematics, the positional coordinates on the screen were extracted from video images recorded by Camera 3. The top, bottom, left, and right edges of the balls and small marks immediately after ball release were manually digitized. Angular velocity was calculated using the method proposed by Jinji and Sakurai (23) and resolved into three components: back/top spin component ($\omega_{x\_0}$), gyro spin component ($\omega_{y\_0}$), and sidespin component ($\omega_{z\_0}$).

**Figure 2:**
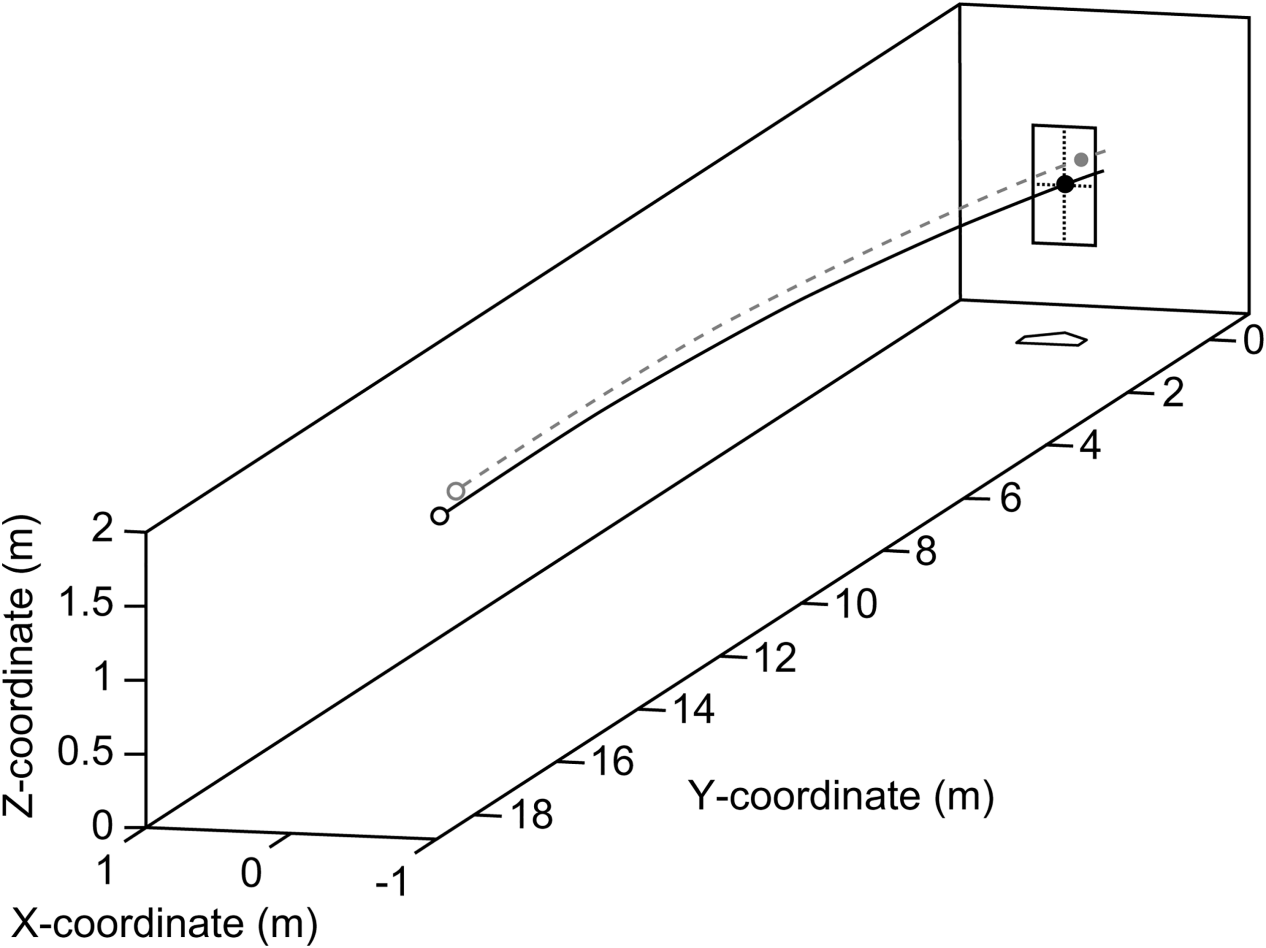
Simulated pitched ball trajectory. The gray dotted line shows the trajectory simulated by the given position, linear velocity, and angular velocity. The black solid line shows the trajectory passing through the center of the strike zone after a coordinate transformation.

**Figure 3:**
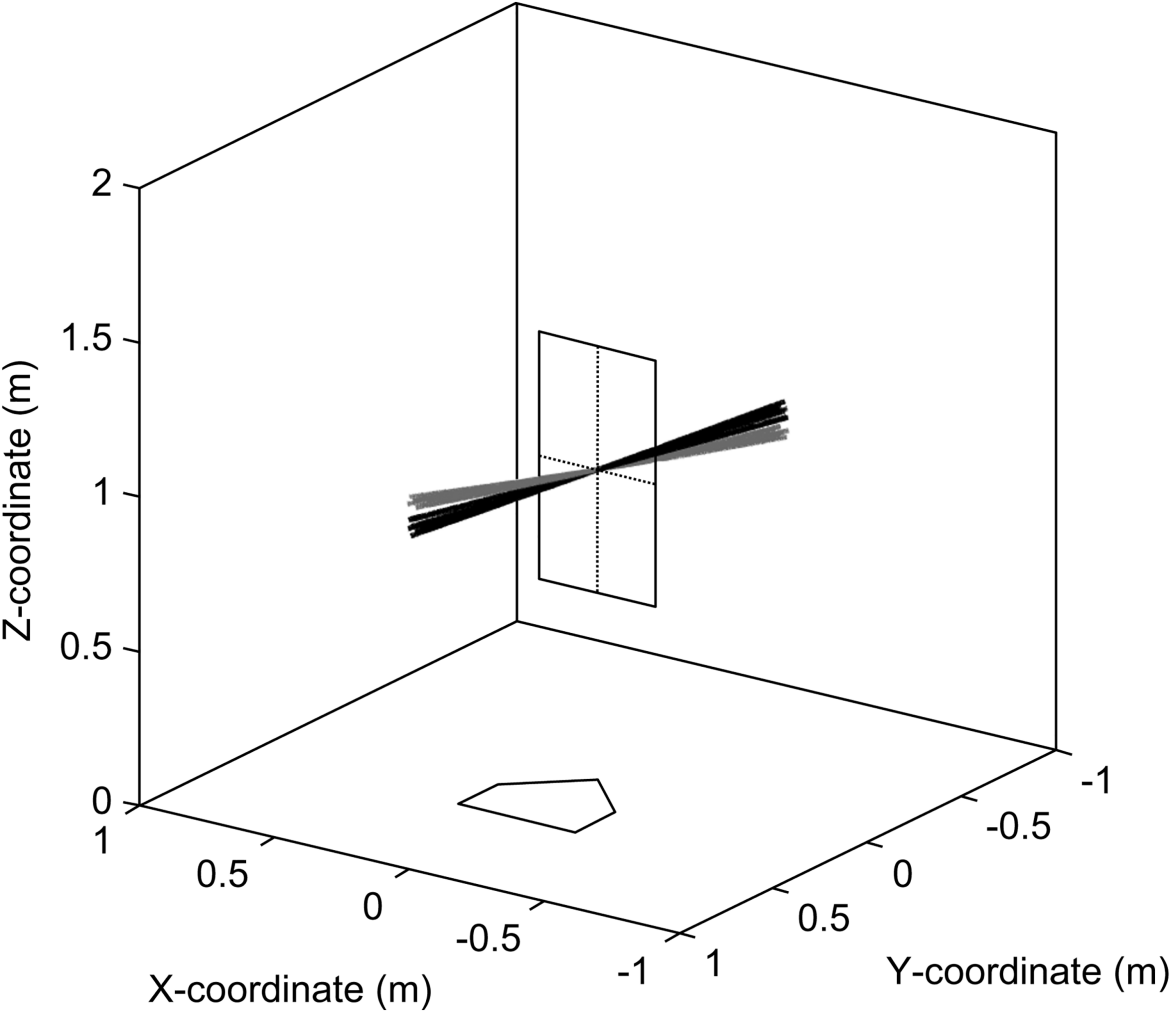
Pitched ball trajectories near the home plate. The black and gray lines show the trajectories of fastballs and curveballs, respectively.

The pitched ball trajectories were simulated using the equations of motion, incorporating the release position ($x{\_}_{0}$, $y{\_}_{0}$, $z{\_}_{0}$), linear velocity ($v_{x\_0}$, $v_{y\_0}$, $v_{z\_0}$), and angular velocity ($\omega_{x\_0}$, $\omega_{y\_0}$, $\omega_{z\_0}$) (Figure 2). Simulation parameters, such as the mass, size, air density, drag coefficient, and lift coefficient, were based on the method described by Kimura et al. (24). The time-step size for simulations was set at 1/50,000 s. Since the simulated pitch trajectory did not always pass through the center of the strike zone (Figure 2, gray dot line), the trajectory was translated to align with the strike zone center at 1 m above the origin (0 m, 0 m, 1 m) (Figure 2, black solid line). For subsequent analyses, only trajectory data with Y-coordinates within the range of 1 m to −1 m were used (Figure 3).

To show the characteristics of the pitched ball in this study, the average, standard deviation, maximum, and minimum pitched ball speed, vertical approach angle, and horizontal approach angle are listed in Table 1. The vertical approach angle was defined as the angle between the pitched ball velocity vector in YZ-plane and -Y. When the pitched ball velocity vector is directed downward, the vertical approach angle is negative. The horizontal approach angle was defined as the angle between the pitched ball velocity vector in XY-plane and -Y. In the case of pitched ball velocity vector directed to the left-handed batter's box, the horizontal approach angle was positive.

**Table 1:** Pitched ball data.

Variables	Pitch type	Average and SD	Maximum	Minimum
Pitched ball speed	Fastball	37.1 ± 1.7 m/s	39.6 m/s	34.9 m/s
	Curveball	28.8 ± 1.5 m/s	31.0 m/s	26.5 m/s
Vertical approach angle	Fastball	−6.3 ± 0.9 deg.	−7.5 deg.	−4.7 deg.
	Curveball	−10.8 ± 0.7 deg.	−11.7 deg.	−9.7 deg.
Horizontal approach angle	Fastball	0.9 ± 1.0 deg.	2.9 deg.	−0.2 deg.
	Curveball	1.2 ± 0.8 deg.	2.6 deg.	0.0 deg.

### 2.3 Bat swing data

#### 2.3.1 Participants

Twenty-nine collegiate baseball batters (height: 173.2 ± 5.6 cm, body mass: 70.6 ± 6.8 kg, age: 19.9 ± 1.3 years) participated in this study. Among them, 15 were right-handed and 14 were left-handed batters.

#### 2.3.2 Experimental procedure

Experiments were conducted in a laboratory. After a sufficient warm-up, participants performed five swings without hitting the ball. They were instructed to swing with maximum effort, simulating a swing at a ball in the center of the strike zone.

#### 2.3.3 Data collection

Retro-reflective markers were attached to the bat head and grip end. The trajectories of these markers were recorded using a motion capture system (MX Giganet, Vicon Motion Systems, UK) with ten cameras (MX T20-S, Vicon Motion Systems) operating at a frame rate of 500 fps. The global coordinate system was defined as follows: the X-axis extended horizontally from the right-handed batter's box to the left-handed batter's box, the Y-axis extended horizontally from the catcher to the pitcher, and the Z-axis represented the vertical direction. The origin of this coordinate system was located at the tip of the home plate. This coordinate system was consistent with the one used to acquire the pitching data.

#### 2.3.4 Data processing

**Figure 4:**
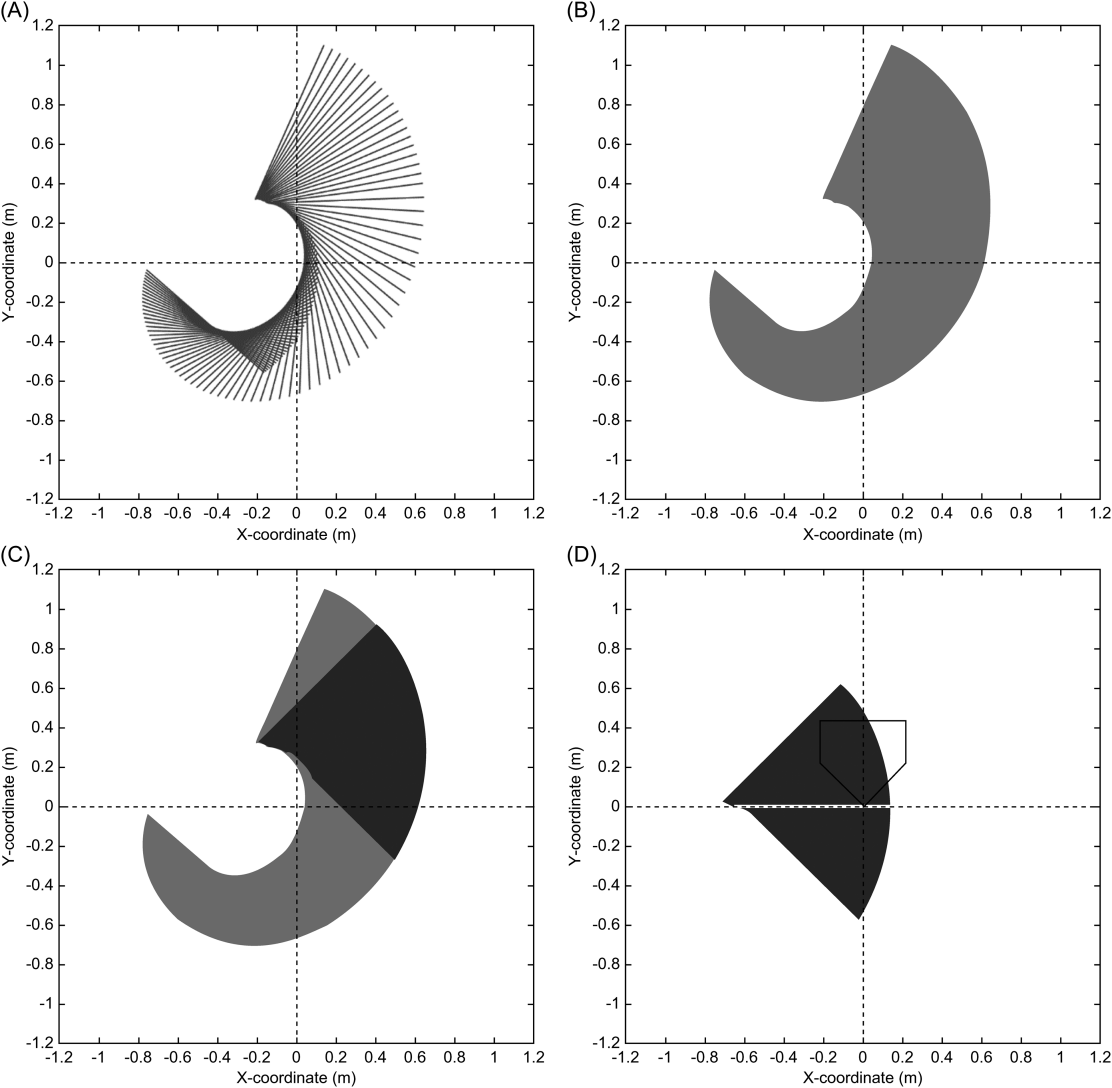
Processing the batting data. **(A)** Bat swing trajectory in the XY-plane at 500 fps. **(B)** Bat swing trajectory with complemented at 50,000 fps. **(C)** Bat swing trajectory with analysis area filled in black. **(D)** Bat swing trajectory translated such that the center of the bat's sweet spot passes through the center of the strike zone.

**Figure 5:**
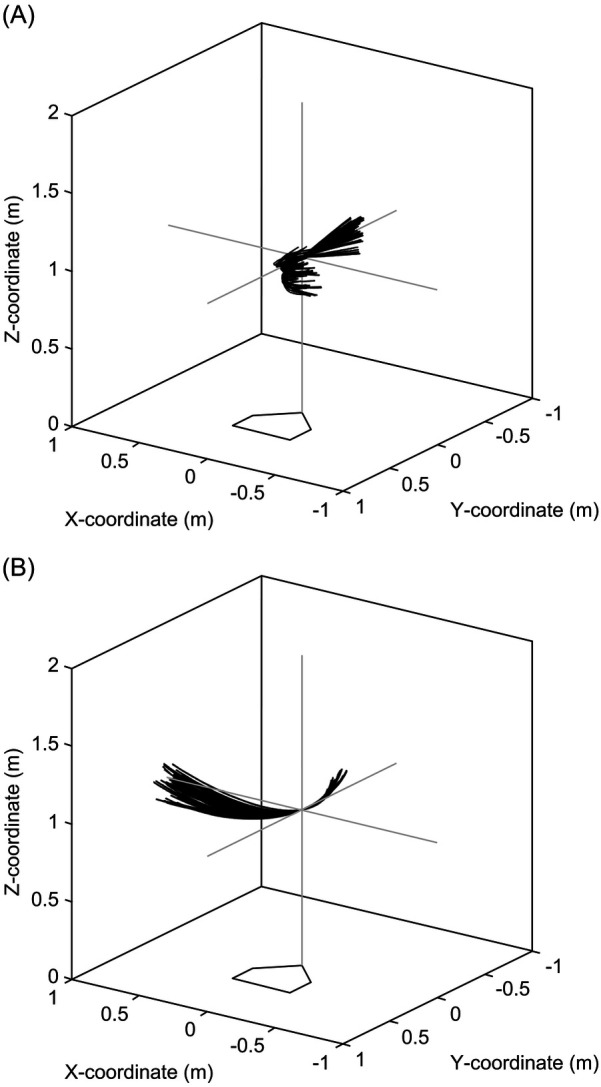
Trajectories of the center of bat sweet spot for **(A)** right-handed batters and **(B)** left-handed batters after a coordinate transformation.

Smoothing was not applied in advance to the marker coordinates of the bat head and grip end to enable spline interpolation, as explained later. An example of a bat swing trajectory in the XY-plane is shown in Figure 4A. Using spline interpolation, the bat swing trajectory data (originally captured at 500 fps) were enhanced at 50,000 fps for the bat head and grip end coordinates (Figure 4B). The rationale for this interpolation will be discussed later section. The horizontal bat angle, defined as the angle between the X-axis and the vector from the bat grip to the bat head in the XY-plane, within a range of ± 45° was used for later analysis (Figure 4C, black area). Since the bat's sweet spot center did not always pass through the strike zone's center, the swing trajectory was adjusted so that the sweet spot center crossed the strike zone's center at the height of 1 m above the origin (0 m, 0 m, 1 m) when the horizontal bat angle was 0° (Figure 4D). Notably, the center of the bat's sweet spot was defined as 15 cm from the bat's head along the bat's long axis. Figure 5 depicts the trajectories of the center of the bat sweet spot for the analysis range of both right- and left-handed batters.

**Figure 6:**
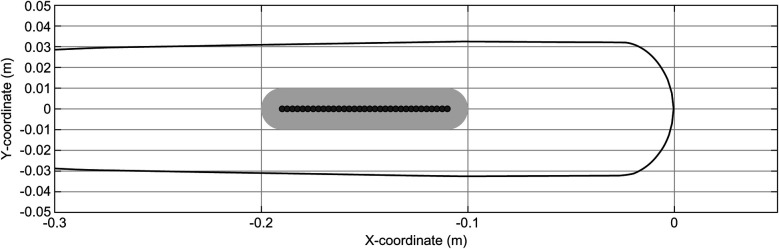
Points produced on the bat's long axis (dark gray dots) and area of the bat's sweet spot (light gray area).

In addition, 800 points were uniformly distributed in a straight line every 0.1 mm along the centerline of the bat's long axis between 11 and 19 cm from the bat head (Figure 6, dark gray dots) for subsequent analysis.

### 2.4 Data analysis

#### 2.4.1 Acceptable range of timing error

The acceptable time and distance range of timing error, indicating that the ball impacted by sweet spot of the bat, were quantified using the pitching and bat swing data.

The bat's sweet spot is not only defined by its length but also by its width. A previous study reported that the bat sweet spot was approximately 10 cm along the long axis and 2 cm along the short axis (10). In our study, the range of the bat sweet spot was defined as an area within 1 cm of any of the 800 points created along the bat's long axis, that is, a rounded rectangle with a 10 cm length and 2 cm short axis, as shown in the light gray area in Figure 6.

**Figure 7:**
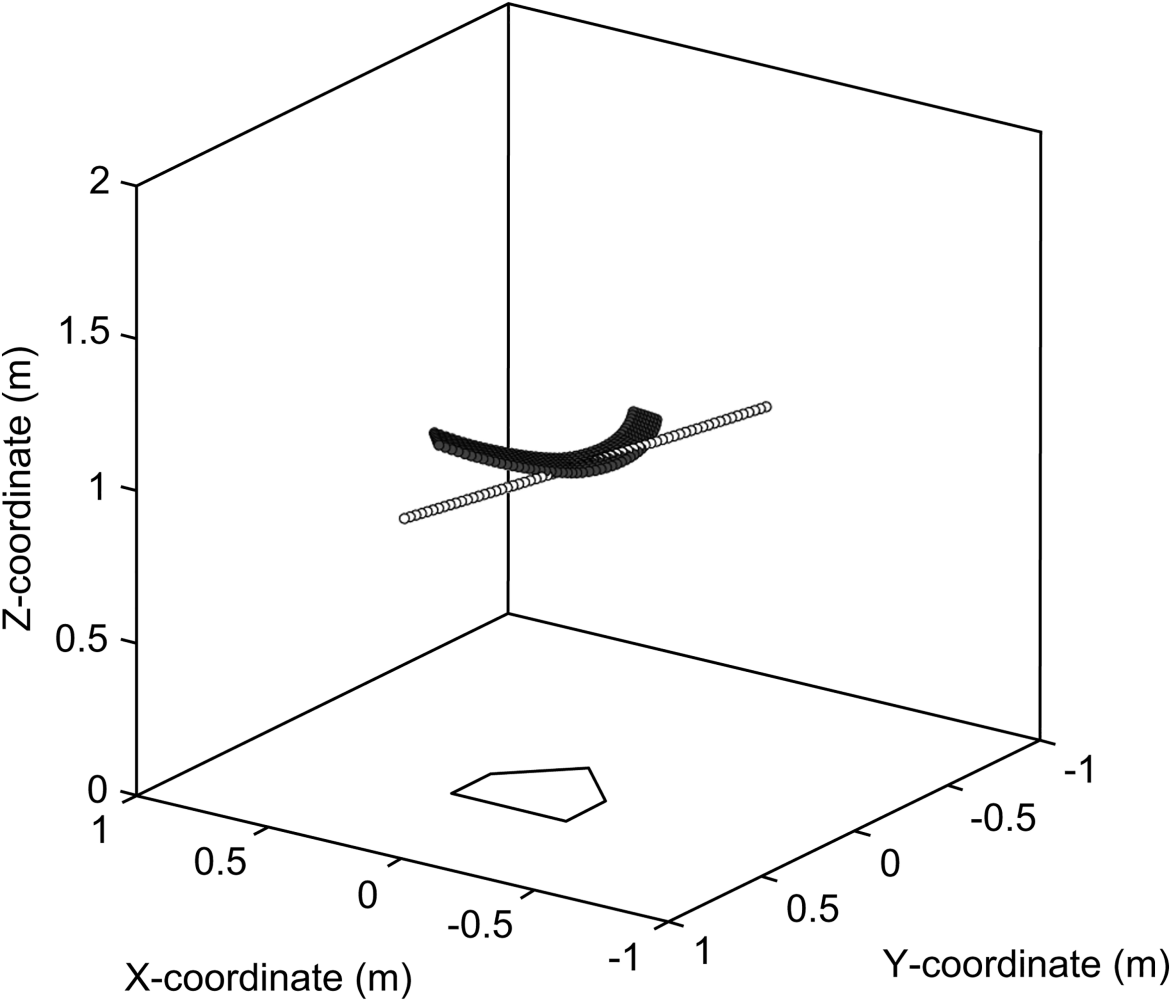
Time-series pitching coordinates (white dots) and point cloud data of the bat sweet spot (gray dots).

The distance between each time-series data of the pitched balls coordinates (Figure 7, white dots) and all points within the bat sweet spot during the swing (Figure 7, gray dots) was calculated. If any point within the bat sweet spot was within 1 cm of the pitched ball coordinate, the ball was considered to be impacted at bat sweet spot. Pitched ball coordinates that were able to impact the area shown in the light gray area in Figure 6 were extracted, and the time and distance from the first to last pitched ball coordinates that were able to impact at bat sweet spot were computed. This process was repeated for 18 pitches multiplied by 145 swings, resulting in 2610 data sets.

The maximum pitched ball speed in our study was 39.6 m/s (Table 1). This translates to simulated pitched ball coordinates at intervals of < 0.79 mm, given a simulation time resolution of 1/50,000 s. The maximum bat head speed in our study was 38.9 m/s (Table 2). This translates to simulated coordinates of the bat head and grip end at intervals of <0.78 mm due to the interpolation of bat coordinates to 1/50,000 s. In summary, potential errors in our method are estimated to be within a range of <0.8 mm, which we consider negligible.

**Table 2:** Bat swing data.

Variables	Batting side	Average and SD	Maximum	Minimum
Bat head speed	Right-handed batter	34.3 ± 2.2 m/s	38.9 m/s	30.7 m/s
	Left-handed batter	34.7 ± 1.9 m/s	37.8 m/s	30.9 m/s
$\theta_{up\;\&\;down}$	Right-handed batter	−6.2 ± 5.5 deg.	12.1 deg.	−19.7 deg.
	Left-handed batter	−4.4 ± 4.1 deg.	6.7 deg.	−14.5 deg.
$\theta_{right\;\&\;left}$	Right-handed batter	6.3 ± 2.6 deg.	12.4 deg.	−3.1 deg.
	Left-handed batter	7.1 ± 3.2 deg.	16.2 deg.	−0.8 deg.
$\theta_{v\_bat}$	Right-handed batter	−26.1 ± 8.5 deg.	−43.2 deg.	−8.4 deg.
	Left-handed batter	−27.6 ± 8.4 deg.	−45.6 deg	−7.4 deg.

#### 2.4.2 Bat swing characteristics

**Figure 8:**
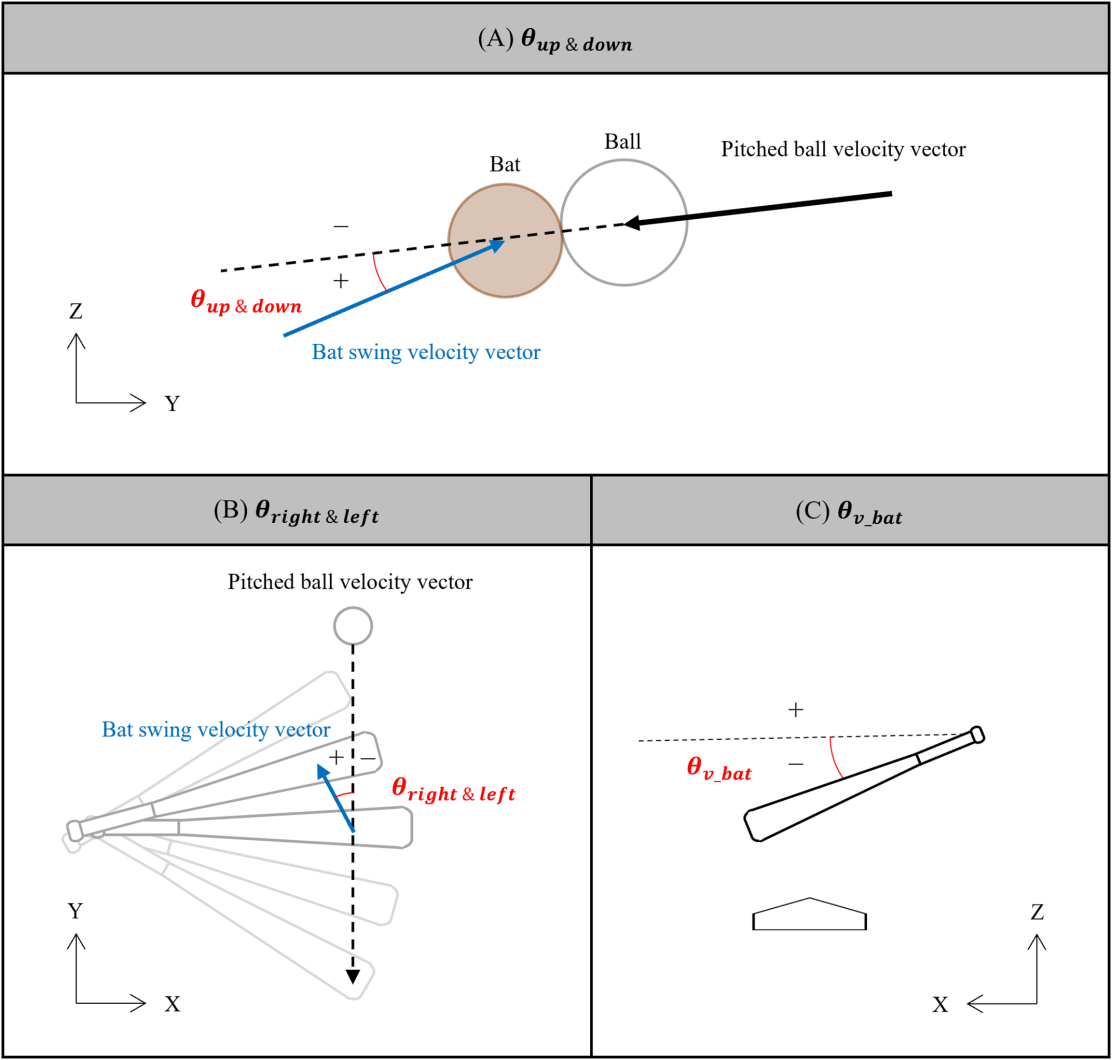
Definition of **(A)** $\theta_{up\;\&\;down}$, **(B)** $\theta_{right\;\&\;left}$, and **(C)** $\theta_{v\_bat}$.

This study quantified bat swing characteristics believed to influence the acceptable range of timing errors. The bat swing angles relative to the pitched balls, $\theta_{up\;\&\;down}$ and $\theta_{left\;\&\;right}$ were defined as the angles of the bat swing velocity vector relative to the pitched ball trajectory at bat-ball impact in the YZ-plane and XY-plane, respectively (Figures 8A,B). The sign of $\theta_{left\;\&\;right}$ was adjusted for right-handed batters. In addition, the vertical bat angle $\theta_{v\_bat}$ was defined as the angle between the bat's long axis at impact and the horizontal plane (Figure 8C). To show the bat swing characteristics in this study, the average, standard deviation, maximum, and minimum bat swing speed, $\theta_{up\;\&\;down}$, $\theta_{left\;\&\;right}$, and $\theta_{v\_bat}$ are listed in Table 2.

### 2.5 Data presentation

The data presentation in this study addressed two primary objectives. For the first objective, the basic statistics of the acceptable time and distance ranges of timing errors were described. For the second objective, the study examined the relationship between $\theta_{up\;\&\;down}$ and the acceptable distance range of the timing error. Focusing on the distance range instead of time eliminates the influence of pitched ball and bat swing speed, allowing for a more accurate analysis. As highlighted in the introduction, factors beyond $\theta_{up\;\&\;down}$ contribute to the acceptable range of timing errors. To clarify why the acceptable range of timing error may become shorter even though the trajectory of the pitch and swing match when viewed from the side, data were extracted where the pitched ball trajectory and bat sweet spot trajectory were aligned within ±3° of $\theta_{up\;\&\;down}$. Furthermore, the relationship of $\theta_{left\;\&\;right}$ and $\theta_{v\_bat}$ with the acceptable range of timing error was investigated. The relationship between $\theta_{left\;\&\;right}$ and acceptable range of timing error was plotted. Plots illustrating these relationships were graded based on the magnitude of $\theta_{v\_bat}$. It should be noted that statistical analysis was not conducted for the second objective. In addition, to provide a clear visual understanding, four typical swing trajectories with varying acceptable ranges of timing errors and swing characteristics were presented.

## 3 Results

**Figure 9:**
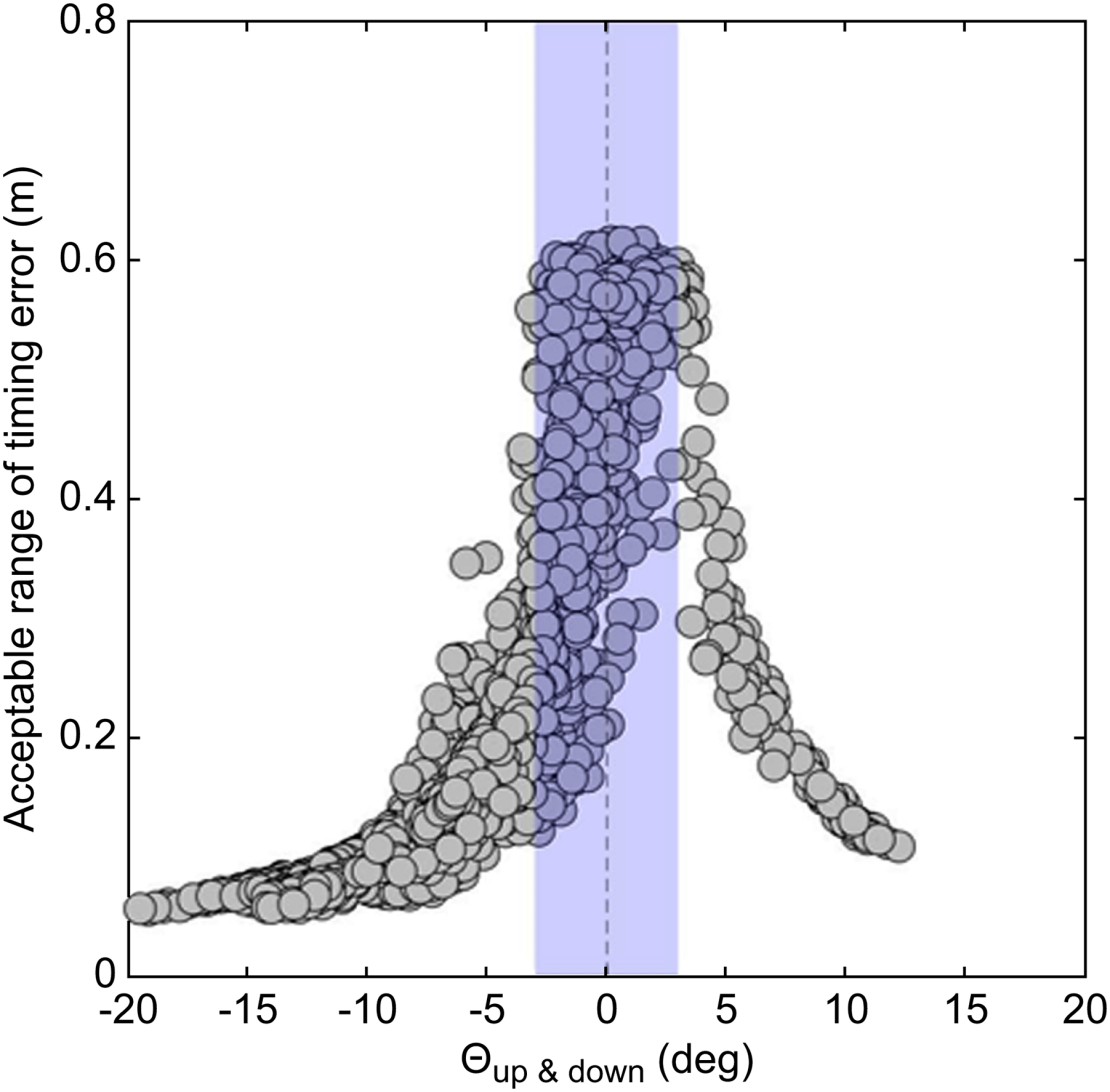
Relationship between $\theta_{up\;\&\;down}$ and the acceptable distance range of the timing error.

**Figure 10:**
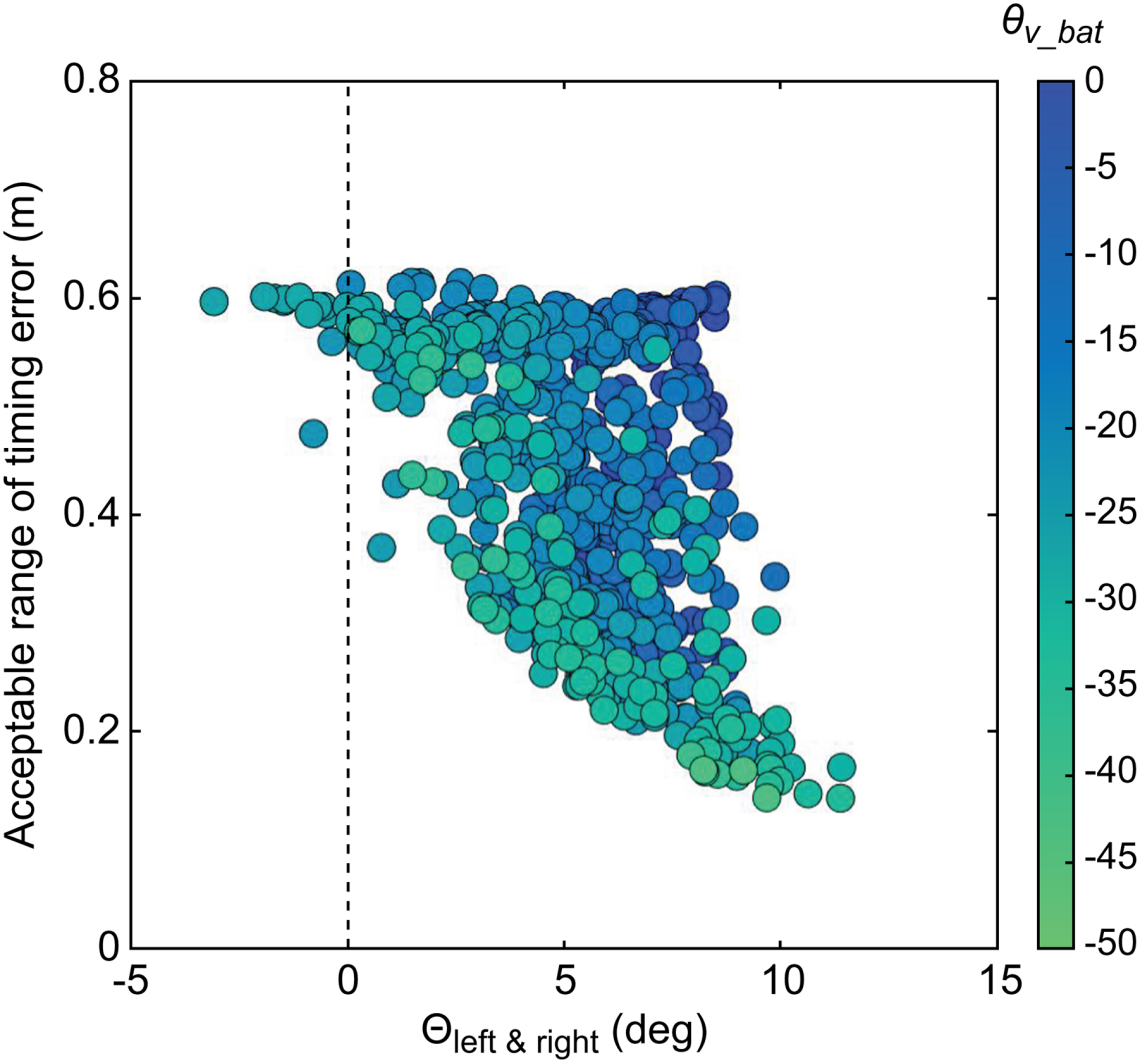
Relationship between $\theta_{right\;\&\;left}$ and the acceptable distance range of the timing error, graded based on the magnitude of $\theta_{v\_bat}$.

**Figure 11:**
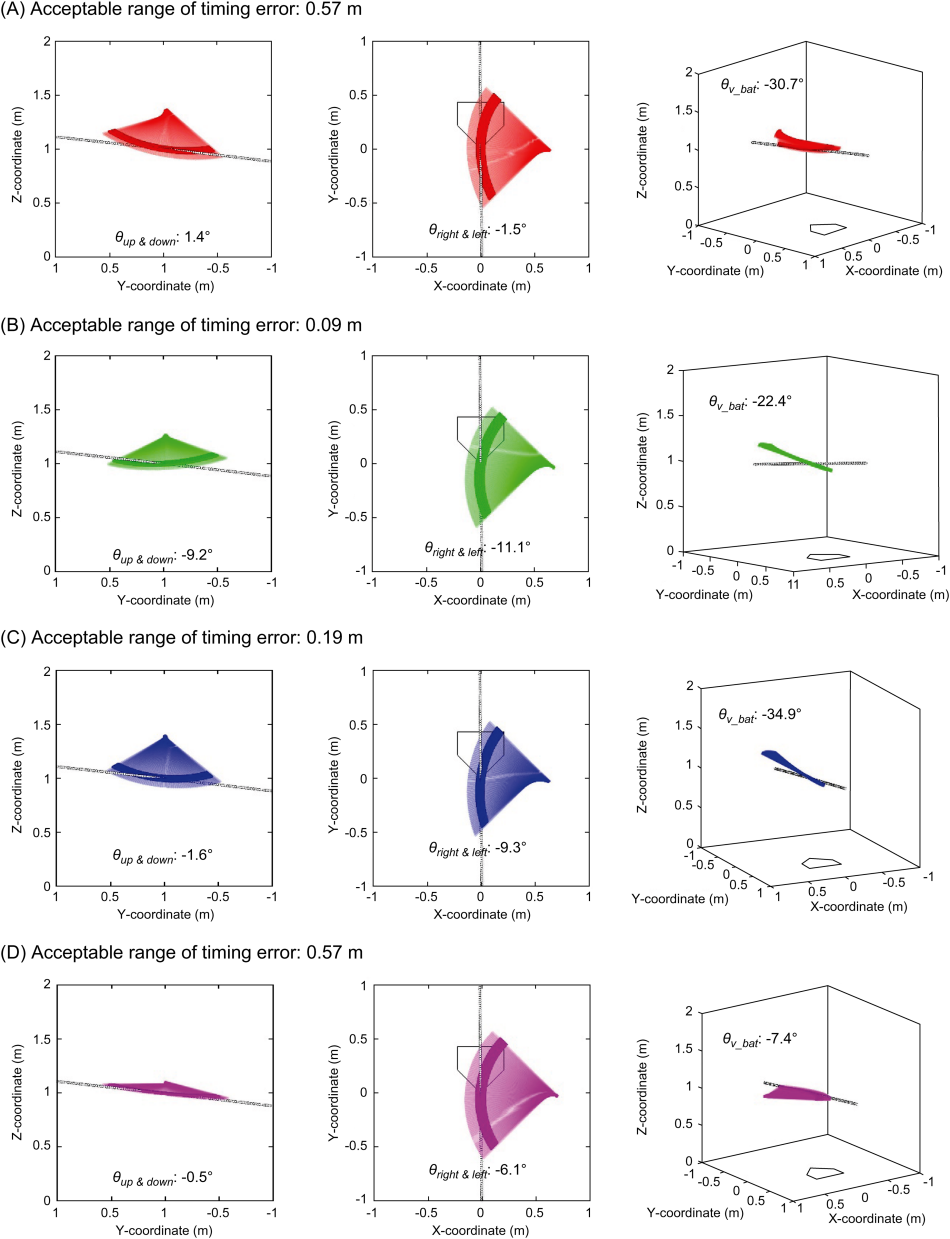
Swing trajectories with different acceptable ranges of timing errors and swing characteristics. **(A)** show the swing with small absolute values of *θ*_*up & down*_ and wide acceptable range of timing error, **(B)** shows the swing with large absolute value of *θ*_*up & down*_ and narrow acceptable range of timing error, **(C)** shows the swing with small absolute value of *θ*_*up & down*_ but narrow acceptable range of timing error because of large absolute value of *θ*_*left & right*_ and largely incline bat, and **(D)** shows the swing with small absolute value of *θ*_*up & down*_ and large absolute value of *θ*_*left & right*_ but wide acceptable range of timing error because of small incline bat.

Table 3 lists the average, standard deviation, maximum, and minimum acceptable time and distance ranges of the timing error. Figure 9 shows the relationship between $\theta_{up\;\&\;down}$ and the acceptable distance range of the timing error. Figure 10 shows the relationship between $\theta_{left\;\&\;right}$ and the acceptable distance range of the timing error, within ±3° of $\theta_{up\;\&\;down}$. The color scale is used to visualize the magnitude of $\theta_{v\_bat}$, with more green colors indicating a greater inclination of the bat. Figures 11A–D illustrate the swing trajectories of different players against the same pitch trajectory, highlighting the varying acceptable ranges of timing errors and swing characteristics.

**Table 3:** Results of the acceptable time and distance range of timing error.

Varibales	Average and SD	Maximum	Minimum
Acceptable time range of timing error	9.36 ± 6.25 ms	30.40 ms	2.48 ms
Acceptable distance range of timing error	0.227 ± 0.163 m	0.614 m	0.056 m

## 4 Discussion

To the best of our knowledge, this is the ﬁrst study to quantify the acceptable time and distance ranges of the timing error based on bat and ball trajectories. The main ﬁndings of our study are twofold. First, it was demonstrated how long and how far the acceptable range of timing error can be widened (maximum time and distance range were 30.40 ms and 0.614 m, respectively). Second, the swing characteristics that influence the acceptable range of timing error were identified.

### 4.1 Acceptable range of timing error

Regarding the accuracy of our proposed method, the average acceptable range of timing error in this study was 9.36 ± 6.25 ms. A previous study reported that the acceptable range of timing error to hit the ball in each direction, 30° left and right around the centerline, was approximately 10 ms when hitting the ball from the pitching machine (17). Therefore, the acceptable range of timing errors calculated in this study is considered a reasonable value compared to the actual batting situation.

Regarding the significance of the acceptable range of timing error, the maximum and minimum acceptable ranges of timing error in the present study were 30.40 ms and 2.48 ms, respectively. This result indicates that the acceptable range of timing error depends on swing characteristics. We explain the extent of the difference based on the pitched ball speed and the time taken for the ball to reach home plate. A pitched ball traveling at 41.7 m/s (150 km/h) reaches the home plate in 420 ms, whereas a ball traveling at 38.9 m/s (140 km/h) takes 450 ms. A player with a sufficiently wide acceptable range of timing error (that is, 30.40 ms) can theoretically contact the ball at the bat's sweet spot for both 150 km/h and 140 km/h pitches using the same swing because the time difference of the pitches is within the acceptable range of timing error. In contrast, a player with a narrow acceptable range of timing error (that is, 2.48 ms) is unable to misjudge the pitched ball speed. In other words, an appropriate swing trajectory for the acceptable range of timing error can compensate for misjudgment of the pitched ball speed of approximately ±5 km/h at most. In addition, timing errors are fundamentally unavoidable in baseball batting. Kidokoro et al. (25) conducted an actual batting experiment using a pitching machine. They found that participants exhibited an average timing error of approximately 10 ms when facing fastballs without prior knowledge of pitch type. Even when given prior information, a timing error of 5 ms persisted. Therefore, it is clear that there is a significant difference in the proportion of missed shots due to timing errors between players with a wide acceptable range of timing errors and those with a narrow range. Given the aforementioned reasons, widening the acceptable range of timing error is crucial for enhancing batting performance.

### 4.2 Relationship between swing characteristics and acceptable range of timing error

What swing characteristics contribute to the wider acceptable range of timing errors? Figure 9 illustrates the relationship between $\theta_{up\;\&\;down}$ and the acceptable distance range of the timing error. As suggested in practical fields (18, 19), it was demonstrated that aligning the trajectory of a bat's sweet spot with the pitched ball trajectory when viewed from the side—specifically, having a small absolute value of $\theta_{up\;\&\;down}$—leads to a wider acceptable range of timing error. For visual clarity, Figures 11A,B illustrate this relationship. Figure 11A illustrates a swing with a small absolute value of $\theta_{up\;\&\;down}$ and a wide acceptable range of timing error, while Figure 11B depicts the opposite. These results confirmed that $\theta_{up\;\&\;down}$ is one of the key factors determining the acceptable range of timing errors.

However, as indicated by the blue section of Figure 9, a smaller absolute value of $\theta_{up\;\&\;down}$ does not necessarily correspond to a wider acceptable range of timing errors. A smaller absolute value of $\theta_{up\;\&\;down}$ is a necessary but not sufficient condition for achieving a wide acceptable range. To analyze this, data within ± 3^∘^ of $\theta_{up\;\&\;down}$ (blue section in Figure 9) were extracted. The relationships between $\theta_{left\;\&\;right}$ and the acceptable range of timing error, categorized by the magnitude of $\theta_{v\_bat}$, are shown in Figure 10. It was revealed that a smaller absolute value of $\theta_{left\;\&\;right}$ corresponded to a wider acceptable range of timing error. That is to say, when both $\theta_{up\;\&\;down}$ and $\theta_{left\;\&\;right}$ are small, meaning the trajectories of the pitched ball and the bat's sweet spot are aligned three-dimensionally, and the acceptable range of timing error is wide (Figure 11A).

On the other hand, when the absolute value of $\theta_{left\;\&\;right}$ is large, the acceptable range of timing error varies, being either wide or narrow. This difference can be attributed to $\theta_{v\_bat}$ as indicated by color-coding. As $\theta_{v\_bat}$ decreased (greener plots in Figure 10), the acceptable range of timing error narrows for a given $\theta_{left\;\&\;right}$. A more inclined bat at the bat-ball impact results in a greater influence of $\theta_{left\;\&\;right}$ on the acceptable range of timing error, leading to a smaller acceptable range. Figures 11C, D illustrate this phenomenon. Both swings have a small absolute value of $\theta_{up\;\&\;down}$ and a large absolute value of $\theta_{left\;\&\;right}$. However, the acceptable range of timing error differs (Figure 11C: narrow, Figure 11D: wide), and this difference is attributed to the magnitude of $\theta_{v\_bat}$.

In summary, the acceptable range of timing error is determined by the combined influence of three variables: $\theta_{up\;\&\;down}$, $\theta_{left\;\&\;right}$, and $\theta_{v\_bat}$. These results highlight the importance of evaluating the three-dimensional alignment of the pitched ball trajectory and the bat's sweet spot when providing instructions aimed at widening the acceptable range of timing errors during practice.

### 4.3 Future work

Future research is expected to clarify the relationship between the acceptable range of timing error and batting performance. In practice, an increased acceptable range of timing error is believed to correlate with a higher probability of the ball making contact with the sweet spot of the bat (18). However, the specific relationship between the magnitude of the acceptable range of timing error and batting accuracy, such as the batting average, has not yet been investigated. Recently, Major League Baseball has tracked the bat swing trajectory and opened it to the public in addition to the pitched ball trajectory (26). In the future, investigating these unresolved issues by using large amounts of data, such as tracking data from the MLB and other organizations, will be necessary.

### 4.4 Limitations

This study had some limitations. First, the acceptable range of timing error obtained in this study was not derived from actual matchups between pitchers and batters. However, this limitation does not diminish the finding that the acceptable range of timing error depended on the swing trajectory. On the other hand, it should be noted that there are inherent limitations to forcibly aligning the pitched ball trajectory with the bat swing trajectory. It is important to acknowledge that this study is based on computational analysis, and that in actual pitcher-batter interactions, both pitched ball and bat swing trajectories vary depending on the pitch's course and height. Second, the batters in this study performed practice swings with maximum effort, assuming that the pitch would be in the center of the strike zone. In real batting situations, batters often adjust their swing trajectories in response to the actual pitched ball trajectory. Consequently, the acceptable range of timing error quantified in this study might have been underestimated. Third, in calculating the acceptable range of timing error, we performed a coordinate transformation such that both the pitch trajectory and swing trajectory passed through the center of the strike zone (0 m, 0 m, 1 m). Consequently, the release point of the pitch was also altered. However, we believe that the pitch data used in this study were not unrealistic because the vertical and horizontal approach angles of the pitched ball remained within the range of previously reported data (27). Fourth, this study did not evaluate whether the batted ball landed in the fair territory. However, in baseball, if a batted ball lands in foul territory, the batter gets another opportunity to hit. Therefore, as a foundational step, this study demonstrated the range within which contact with the bat's sweet spot is possible.

## 5 Conclusion

We quantified the acceptable time and distance ranges of the timing errors based on the trajectories of pitched balls and bat swings. The findings revealed that the average acceptable range of timing error was 9.36 ± 6.25 ms in time and 0.23 ± 0.16 m in distance, and their range was varied (time: 2.48‒30.40 ms, distance: 0.06‒0.61 m) depending on the swing trajectory. Furthermore, the swing characteristics that influence the acceptable range of timing errors were identified. These results highlight the importance of instructing batters to develop swing techniques with a three-dimensional perspective.

## Data Availability

The raw data supporting the conclusions of this article will be made available by the authors, without undue reservation.
